# 1,5-Bis(2-oxoindolin-3-yl­idene)thio­carbonohydrazide tetra­hydro­furan monosolvate

**DOI:** 10.1107/S1600536812022714

**Published:** 2012-05-23

**Authors:** Shaghayegh Pezeshkpour, Hamid Khaledi, Hapipah Mohd Ali

**Affiliations:** a Department of Chemistry, University of Malaya, 50603 Kuala Lumpur, Malaysia

## Abstract

In the thio­carbonohydrazide mol­ecule of the title compound, C_17_H_12_N_6_O_2_S·C_4_H_8_O, the terminal indolin-2-one ring systems make a dihedral angle of 20.13 (6)° with each other. Two intra­molecular N—H⋯O hydrogen bonds are present, each of which generates an *S*(6) ring. In the crystal, N—H⋯O hydrogen bonds lead to a mol­ecular chain running along the *b* axis. The tetra­hydro­furan solvent mol­ecule is disordered over two orientations in a 0.561 (11):0.439 (11) ratio.

## Related literature

For the structures of the *N*-methyl­isatin analogue and its Sn(IV) complex and also the spectroscopic characterization of the title thio­carbonohydrazide, see: Bacchi *et al.* (2005 ▶).
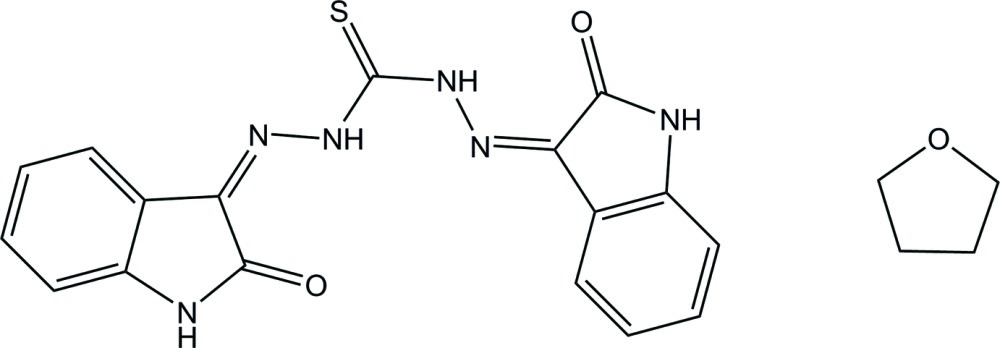


## Experimental

### 

#### Crystal data

C_17_H_12_N_6_O_2_S·C_4_H_8_O
*M* *_r_* = 436.49
Triclinic, 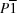

*a* = 8.4768 (1) Å
*b* = 11.4765 (2) Å
*c* = 11.9091 (2) Å
α = 75.206 (1)°
β = 72.553 (1)°
γ = 69.416 (1)°
*V* = 1020.02 (3) Å^3^
*Z* = 2
Mo *K*α radiation
μ = 0.20 mm^−1^
*T* = 296 K
0.33 × 0.25 × 0.13 mm

#### Data collection

Bruker APEXII CCD diffractometer
Absorption correction: multi-scan (*SADABS*; Sheldrick, 1996 ▶) *T*_min_ = 0.938, *T*_max_ = 0.975
9421 measured reflections
4451 independent reflections
3628 reflections with *I* > 2σ(*I*)
*R*_int_ = 0.016

#### Refinement

*R*[*F* ^2^ > 2σ(*F* ^2^)] = 0.040
*wR*(*F* ^2^) = 0.121
*S* = 1.05
4451 reflections
338 parameters
30 restraints
H atoms treated by a mixture of independent and constrained refinement
Δρ_max_ = 0.20 e Å^−3^
Δρ_min_ = −0.28 e Å^−3^

### 

Data collection: *APEX2* (Bruker, 2007 ▶); cell refinement: *SAINT* (Bruker, 2007 ▶); data reduction: *SAINT*; program(s) used to solve structure: *SHELXS97* (Sheldrick, 2008 ▶); program(s) used to refine structure: *SHELXL97* (Sheldrick, 2008 ▶); molecular graphics: *X-SEED* (Barbour, 2001 ▶); software used to prepare material for publication: *SHELXL97* and *publCIF* (Westrip, 2010 ▶).

## Supplementary Material

Crystal structure: contains datablock(s) I, global. DOI: 10.1107/S1600536812022714/is5142sup1.cif

Structure factors: contains datablock(s) I. DOI: 10.1107/S1600536812022714/is5142Isup2.hkl

Supplementary material file. DOI: 10.1107/S1600536812022714/is5142Isup3.cml

Additional supplementary materials:  crystallographic information; 3D view; checkCIF report

## Figures and Tables

**Table 1:** Hydrogen-bond geometry (Å, °)

*D*—H⋯*A*	*D*—H	H⋯*A*	*D*⋯*A*	*D*—H⋯*A*
N1—H1⋯O3^i^	0.88 (1)	1.96 (2)	2.829 (15)	168 (2)
N1—H1⋯O3′^i^	0.88 (1)	1.98 (3)	2.84 (2)	167 (2)
N3—H3⋯O1	0.85 (1)	2.18 (2)	2.8369 (16)	134 (2)
N4—H4⋯O2	0.86 (1)	2.10 (2)	2.7857 (16)	136 (2)
N6—H6⋯O1^ii^	0.84 (1)	2.32 (2)	3.0522 (16)	146 (2)

Symmetry codes: (i) 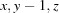; (ii) 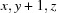.

**Fig. 1.**
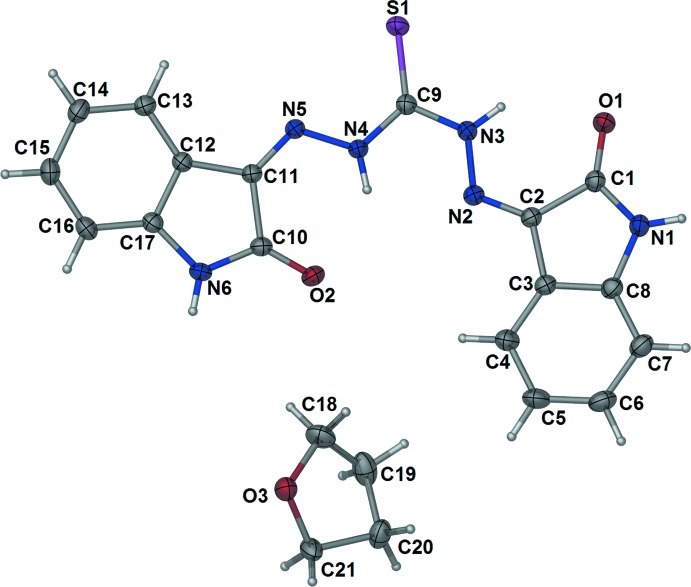
Displacement ellipsoid plot of the title compound at the 30% probability level. Hydrogen atoms are drawn as spheres of arbitrary radius. Only the major component of the disordered tetrahydrofuran molecule is shown.

**Fig. 2.**
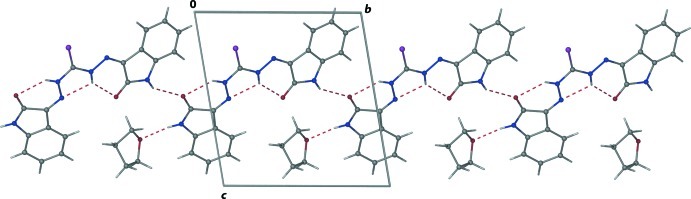
A packing diagram, showing the N—H···O hydrogen bonded chain along the b axis. Hydrogen bonds are depicted as red dashed lines.

## supplementary crystallographic information

### Comment

Recently, a series of isatin-based thiocarbonohydrazides and the related Sn(IV) complexes were synthesized and studied for their antimicrobial and mutagenic properties (Bacchi *et al.*, 2005). The title compound, being among those, was re-synthesized and grown as X-ray quality crystals from THF by our research group. In the crystal structure, the hydrazone molecule is roughly planar with the maximum deviation from the least-squares plane of all non-H atoms being 0.481 (2) Å for atom C14. The configurations around the C—N bonds, stabilized by intramolecular N—H···O hydrogen bonding (Table 1), are same as those observed in the *N*-methylisatin analogous. The hydrazone molecule is co-crystallized with one molecule of THF which suffers from disorder. The crystal packing contains chains along the *b* axis formed by intermolecular N6—H6···O1 hydrogen bonds (Table 1 and Fig. 2). The THF molecules are N—H···O bonded to the chain.

### Experimental

The Schiff base was prepared as described previously (Bacchi *et al.*, 2005) and grown as X-ray quality crystals from a THF solution at room temperature.

### Refinement

C-bound hydrogen atoms were placed at the calculated positions and refined in riding mode with C—H distances of 0.93 Å. The amino hydrogen atoms were located in a difference Fourier map and refined with N—H distance restraints of 0.86 (2) Å. For all the hydrogen atoms *U*_iso_(H) were set to 1.2 *U*_eq_(carrier atoms). The tetrahydrofuran molecule was found to be disordered over two positions, the site occupancy factor for the major component refined to 0.561 (11). The geometrical parameters of the two disordered components were kept similar by using the SAME command in *SHELXL97*.

### Crystal data

**Table:**

C_17_H_12_N_6_O_2_S·C_4_H_8_O	*Z* = 2
*M**_r_* = 436.49	*F*(000) = 456
Triclinic, *P*1	*D*_x_ = 1.421 Mg m^−^^3^
Hall symbol: -P 1	Mo *K*α radiation, λ = 0.71073 Å
*a* = 8.4768 (1) Å	Cell parameters from 4598 reflections
*b* = 11.4765 (2) Å	θ = 2.4–29.1°
*c* = 11.9091 (2) Å	µ = 0.20 mm^−^^1^
α = 75.206 (1)°	*T* = 296 K
β = 72.553 (1)°	Block, yellow
γ = 69.416 (1)°	0.33 × 0.25 × 0.13 mm
*V* = 1020.02 (3) Å^3^	

### Data collection

**Table:**

Bruker APEXII CCD diffractometer	4451 independent reflections
Radiation source: fine-focus sealed tube	3628 reflections with *I* > 2σ(*I*)
Graphite monochromator	*R*_int_ = 0.016
φ and ω scans	θ_max_ = 27.0°, θ_min_ = 1.9°
Absorption correction: multi-scan (*SADABS*; Sheldrick, 1996)	*h* = −10→10
*T*_min_ = 0.938, *T*_max_ = 0.975	*k* = −14→14
9421 measured reflections	*l* = −15→15

### Refinement

**Table:**

Refinement on *F*^2^	Primary atom site location: structure-invariant direct methods
Least-squares matrix: full	Secondary atom site location: difference Fourier map
*R*[*F*^2^ > 2σ(*F*^2^)] = 0.040	Hydrogen site location: inferred from neighbouring sites
*wR*(*F*^2^) = 0.121	H atoms treated by a mixture of independent and constrained refinement
*S* = 1.05	*w* = 1/[σ^2^(*F*_o_^2^) + (0.0692*P*)^2^ + 0.1602*P*] where *P* = (*F*_o_^2^ + 2*F*_c_^2^)/3
4451 reflections	(Δ/σ)_max_ = 0.001
338 parameters	Δρ_max_ = 0.20 e Å^−^^3^
30 restraints	Δρ_min_ = −0.28 e Å^−^^3^

### Special details

**Table:**

Geometry. All e.s.d.'s (except the e.s.d. in the dihedral angle between two l.s. planes) are estimated using the full covariance matrix. The cell e.s.d.'s are taken into account individually in the estimation of e.s.d.'s in distances, angles and torsion angles; correlations between e.s.d.'s in cell parameters are only used when they are defined by crystal symmetry. An approximate (isotropic) treatment of cell e.s.d.'s is used for estimating e.s.d.'s involving l.s. planes.
Refinement. Refinement of *F*^2^ against ALL reflections. The weighted *R*-factor *wR* and goodness of fit *S* are based on *F*^2^, conventional *R*-factors *R* are based on *F*, with *F* set to zero for negative *F*^2^. The threshold expression of *F*^2^ > σ(*F*^2^) is used only for calculating *R*-factors(gt) *etc*. and is not relevant to the choice of reflections for refinement. *R*-factors based on *F*^2^ are statistically about twice as large as those based on *F*, and *R*- factors based on ALL data will be even larger.

### Fractional atomic coordinates and isotropic or equivalent isotropic displacement parameters (Å^2^)

**Table:**

	*x*	*y*	*z*	*U*_iso_*/*U*_eq_	Occ. (<1)
S1	0.05012 (6)	0.21013 (4)	0.21038 (4)	0.05637 (15)	
O1	0.25587 (17)	−0.15327 (11)	0.48629 (10)	0.0564 (3)	
O2	0.20530 (16)	0.44464 (11)	0.49172 (9)	0.0529 (3)	
N1	0.38131 (19)	−0.19240 (13)	0.64563 (12)	0.0498 (3)	
H1	0.402 (2)	−0.2746 (14)	0.6651 (16)	0.060*	
N2	0.21787 (17)	0.11827 (12)	0.49897 (11)	0.0435 (3)	
N3	0.16236 (18)	0.11149 (12)	0.40644 (12)	0.0455 (3)	
H3	0.163 (2)	0.0406 (14)	0.3970 (16)	0.055*	
N4	0.13631 (18)	0.32009 (11)	0.34784 (11)	0.0437 (3)	
H4	0.167 (2)	0.3167 (17)	0.4115 (13)	0.052*	
N5	0.11499 (16)	0.42919 (11)	0.26740 (11)	0.0411 (3)	
N6	0.18651 (18)	0.64925 (12)	0.39333 (11)	0.0470 (3)	
H6	0.217 (2)	0.6750 (18)	0.4406 (15)	0.056*	
C1	0.3000 (2)	−0.11911 (14)	0.55902 (13)	0.0448 (3)	
C2	0.2764 (2)	0.01502 (14)	0.56776 (13)	0.0417 (3)	
C3	0.3419 (2)	0.00786 (15)	0.66947 (13)	0.0442 (3)	
C4	0.3523 (3)	0.09899 (18)	0.72112 (17)	0.0617 (5)	
H4A	0.3114	0.1843	0.6909	0.074*	
C5	0.4251 (3)	0.0596 (2)	0.81909 (18)	0.0709 (5)	
H5	0.4332	0.1193	0.8555	0.085*	
C6	0.4860 (3)	−0.0675 (2)	0.86359 (16)	0.0634 (5)	
H6A	0.5342	−0.0916	0.9297	0.076*	
C7	0.4769 (2)	−0.15979 (17)	0.81209 (14)	0.0542 (4)	
H7	0.5184	−0.2451	0.8421	0.065*	
C8	0.4042 (2)	−0.11990 (15)	0.71482 (13)	0.0441 (3)	
C9	0.1173 (2)	0.21704 (14)	0.32405 (13)	0.0423 (3)	
C10	0.1806 (2)	0.52963 (14)	0.40708 (13)	0.0425 (3)	
C11	0.13649 (18)	0.52272 (13)	0.29545 (12)	0.0388 (3)	
C12	0.12560 (19)	0.64527 (13)	0.21980 (13)	0.0399 (3)	
C13	0.0978 (2)	0.69308 (15)	0.10567 (13)	0.0469 (4)	
H13	0.0746	0.6448	0.0641	0.056*	
C14	0.1054 (2)	0.81390 (16)	0.05535 (15)	0.0558 (4)	
H14	0.0881	0.8473	−0.0213	0.067*	
C15	0.1386 (3)	0.88613 (16)	0.11776 (17)	0.0600 (5)	
H15	0.1432	0.9674	0.0820	0.072*	
C16	0.1649 (2)	0.84010 (15)	0.23188 (16)	0.0544 (4)	
H16	0.1860	0.8891	0.2737	0.065*	
C17	0.1587 (2)	0.71911 (14)	0.28141 (13)	0.0426 (3)	
O3	0.446 (3)	0.5472 (16)	0.7408 (9)	0.054 (3)	0.561 (11)
C18	0.3597 (15)	0.4754 (11)	0.7110 (8)	0.073 (3)	0.561 (11)
H18C	0.4432	0.4071	0.6710	0.087*	0.561 (11)
H18D	0.2833	0.5288	0.6592	0.087*	0.561 (11)
C19	0.2583 (7)	0.4248 (5)	0.8281 (7)	0.0748 (19)	0.561 (11)
H19C	0.2449	0.3451	0.8256	0.090*	0.561 (11)
H19D	0.1447	0.4843	0.8505	0.090*	0.561 (11)
C20	0.3706 (7)	0.4076 (5)	0.9136 (5)	0.0656 (13)	0.561 (11)
H20C	0.3065	0.4015	0.9963	0.079*	0.561 (11)
H20D	0.4731	0.3351	0.9042	0.079*	0.561 (11)
C21	0.4135 (17)	0.5314 (11)	0.8674 (8)	0.062 (2)	0.561 (11)
H21C	0.3172	0.6007	0.8976	0.075*	0.561 (11)
H21D	0.5151	0.5278	0.8914	0.075*	0.561 (11)
O3'	0.471 (3)	0.545 (2)	0.7353 (11)	0.049 (2)	0.439 (11)
C18'	0.3702 (17)	0.5049 (11)	0.6829 (10)	0.058 (2)	0.439 (11)
H18A	0.4341	0.4837	0.6047	0.070*	0.439 (11)
H18B	0.2623	0.5705	0.6754	0.070*	0.439 (11)
C19'	0.3368 (12)	0.3903 (7)	0.7693 (8)	0.078 (2)	0.439 (11)
H19A	0.4385	0.3170	0.7618	0.094*	0.439 (11)
H19B	0.2398	0.3711	0.7591	0.094*	0.439 (11)
C20'	0.2948 (18)	0.4357 (11)	0.8886 (8)	0.109 (4)	0.439 (11)
H20A	0.1763	0.4895	0.9079	0.131*	0.439 (11)
H20B	0.3140	0.3652	0.9535	0.131*	0.439 (11)
C21'	0.4234 (19)	0.5088 (14)	0.8621 (11)	0.062 (3)	0.439 (11)
H21A	0.3714	0.5832	0.9000	0.074*	0.439 (11)
H21B	0.5249	0.4567	0.8922	0.074*	0.439 (11)

### Atomic displacement parameters (Å^2^)

**Table:**

	*U*^11^	*U*^22^	*U*^33^	*U*^12^	*U*^13^	*U*^23^
S1	0.0781 (3)	0.0465 (2)	0.0592 (3)	−0.0176 (2)	−0.0398 (2)	−0.00685 (18)
O1	0.0811 (8)	0.0489 (6)	0.0550 (7)	−0.0291 (6)	−0.0329 (6)	−0.0013 (5)
O2	0.0720 (8)	0.0532 (6)	0.0403 (6)	−0.0235 (6)	−0.0238 (5)	0.0002 (5)
N1	0.0666 (9)	0.0398 (7)	0.0513 (8)	−0.0214 (6)	−0.0276 (7)	0.0027 (6)
N2	0.0528 (7)	0.0407 (6)	0.0406 (6)	−0.0147 (5)	−0.0171 (5)	−0.0045 (5)
N3	0.0621 (8)	0.0359 (6)	0.0462 (7)	−0.0167 (6)	−0.0251 (6)	−0.0024 (5)
N4	0.0616 (8)	0.0368 (6)	0.0390 (6)	−0.0157 (6)	−0.0221 (6)	−0.0031 (5)
N5	0.0518 (7)	0.0361 (6)	0.0380 (6)	−0.0123 (5)	−0.0171 (5)	−0.0041 (5)
N6	0.0634 (8)	0.0460 (7)	0.0417 (7)	−0.0197 (6)	−0.0196 (6)	−0.0108 (5)
C1	0.0539 (9)	0.0416 (8)	0.0438 (8)	−0.0205 (7)	−0.0171 (7)	0.0002 (6)
C2	0.0484 (8)	0.0412 (7)	0.0386 (7)	−0.0162 (6)	−0.0132 (6)	−0.0042 (6)
C3	0.0511 (8)	0.0457 (8)	0.0376 (7)	−0.0151 (6)	−0.0140 (6)	−0.0046 (6)
C4	0.0863 (13)	0.0511 (9)	0.0559 (10)	−0.0162 (9)	−0.0307 (9)	−0.0118 (8)
C5	0.1002 (16)	0.0693 (12)	0.0592 (11)	−0.0239 (11)	−0.0358 (11)	−0.0190 (9)
C6	0.0763 (12)	0.0757 (12)	0.0461 (9)	−0.0234 (10)	−0.0281 (9)	−0.0061 (8)
C7	0.0612 (10)	0.0577 (10)	0.0451 (8)	−0.0196 (8)	−0.0212 (7)	0.0023 (7)
C8	0.0474 (8)	0.0483 (8)	0.0396 (7)	−0.0197 (7)	−0.0124 (6)	−0.0017 (6)
C9	0.0478 (8)	0.0392 (7)	0.0431 (8)	−0.0128 (6)	−0.0159 (6)	−0.0062 (6)
C10	0.0492 (8)	0.0453 (8)	0.0374 (7)	−0.0155 (6)	−0.0140 (6)	−0.0080 (6)
C11	0.0450 (8)	0.0382 (7)	0.0350 (7)	−0.0121 (6)	−0.0124 (6)	−0.0063 (5)
C12	0.0451 (8)	0.0370 (7)	0.0387 (7)	−0.0116 (6)	−0.0115 (6)	−0.0069 (6)
C13	0.0564 (9)	0.0452 (8)	0.0407 (8)	−0.0138 (7)	−0.0158 (7)	−0.0065 (6)
C14	0.0693 (11)	0.0483 (9)	0.0450 (8)	−0.0145 (8)	−0.0188 (8)	0.0026 (7)
C15	0.0777 (12)	0.0387 (8)	0.0625 (10)	−0.0189 (8)	−0.0206 (9)	0.0008 (7)
C16	0.0696 (11)	0.0410 (8)	0.0587 (10)	−0.0185 (7)	−0.0184 (8)	−0.0118 (7)
C17	0.0483 (8)	0.0388 (7)	0.0423 (8)	−0.0110 (6)	−0.0130 (6)	−0.0092 (6)
O3	0.066 (6)	0.049 (3)	0.053 (3)	−0.023 (4)	−0.019 (2)	−0.003 (2)
C18	0.085 (4)	0.078 (7)	0.080 (4)	−0.035 (4)	−0.037 (3)	−0.019 (4)
C19	0.059 (3)	0.053 (2)	0.115 (5)	−0.020 (2)	−0.028 (3)	−0.005 (3)
C20	0.070 (3)	0.053 (2)	0.073 (3)	−0.0203 (19)	−0.032 (2)	0.0115 (18)
C21	0.098 (6)	0.048 (3)	0.041 (3)	−0.029 (4)	−0.004 (3)	−0.012 (2)
O3'	0.055 (5)	0.054 (4)	0.047 (4)	−0.015 (2)	−0.021 (3)	−0.014 (3)
C18'	0.057 (4)	0.047 (4)	0.080 (5)	−0.012 (3)	−0.029 (4)	−0.016 (4)
C19'	0.072 (4)	0.057 (3)	0.112 (5)	−0.028 (3)	−0.014 (4)	−0.022 (3)
C20'	0.152 (11)	0.123 (8)	0.079 (5)	−0.090 (8)	−0.025 (6)	0.009 (5)
C21'	0.066 (5)	0.059 (6)	0.059 (5)	−0.005 (3)	−0.016 (3)	−0.023 (3)

### Geometric parameters (Å, º)

**Table:**

S1—C9	1.6467 (15)	C13—H13	0.9300
O1—C1	1.2307 (18)	C14—C15	1.387 (3)
O2—C10	1.2215 (18)	C14—H14	0.9300
N1—C1	1.350 (2)	C15—C16	1.381 (3)
N1—C8	1.403 (2)	C15—H15	0.9300
N1—H1	0.877 (14)	C16—C17	1.376 (2)
N2—C2	1.2868 (19)	C16—H16	0.9300
N2—N3	1.3498 (17)	O3—C21	1.424 (9)
N3—C9	1.3670 (19)	O3—C18	1.438 (9)
N3—H3	0.847 (14)	C18—C19	1.496 (9)
N4—C9	1.3557 (19)	C18—H18C	0.9700
N4—N5	1.3601 (17)	C18—H18D	0.9700
N4—H4	0.860 (14)	C19—C20	1.527 (6)
N5—C11	1.2861 (18)	C19—H19C	0.9700
N6—C10	1.357 (2)	C19—H19D	0.9700
N6—C17	1.4088 (19)	C20—C21	1.516 (9)
N6—H6	0.837 (14)	C20—H20C	0.9700
C1—C2	1.508 (2)	C20—H20D	0.9700
C2—C3	1.450 (2)	C21—H21C	0.9700
C3—C4	1.380 (2)	C21—H21D	0.9700
C3—C8	1.394 (2)	O3'—C21'	1.430 (11)
C4—C5	1.383 (3)	O3'—C18'	1.438 (11)
C4—H4A	0.9300	C18'—C19'	1.504 (9)
C5—C6	1.384 (3)	C18'—H18A	0.9700
C5—H5	0.9300	C18'—H18B	0.9700
C6—C7	1.386 (3)	C19'—C20'	1.536 (10)
C6—H6A	0.9300	C19'—H19A	0.9700
C7—C8	1.375 (2)	C19'—H19B	0.9700
C7—H7	0.9300	C20'—C21'	1.515 (11)
C10—C11	1.5123 (19)	C20'—H20A	0.9700
C11—C12	1.4525 (19)	C20'—H20B	0.9700
C12—C13	1.387 (2)	C21'—H21A	0.9700
C12—C17	1.394 (2)	C21'—H21B	0.9700
C13—C14	1.379 (2)		
			
C1—N1—C8	111.71 (13)	C14—C15—H15	119.3
C1—N1—H1	124.8 (13)	C17—C16—C15	117.62 (15)
C8—N1—H1	122.7 (13)	C17—C16—H16	121.2
C2—N2—N3	118.31 (12)	C15—C16—H16	121.2
N2—N3—C9	119.91 (12)	C16—C17—C12	121.62 (14)
N2—N3—H3	119.6 (12)	C16—C17—N6	128.80 (14)
C9—N3—H3	120.3 (12)	C12—C17—N6	109.57 (12)
C9—N4—N5	119.78 (12)	C21—O3—C18	108.8 (8)
C9—N4—H4	121.0 (12)	O3—C18—C19	104.9 (7)
N5—N4—H4	119.1 (12)	O3—C18—H18C	110.8
C11—N5—N4	116.37 (12)	C19—C18—H18C	110.8
C10—N6—C17	111.43 (12)	O3—C18—H18D	110.8
C10—N6—H6	124.2 (13)	C19—C18—H18D	110.8
C17—N6—H6	123.8 (13)	H18C—C18—H18D	108.8
O1—C1—N1	127.78 (14)	C18—C19—C20	102.6 (5)
O1—C1—C2	126.52 (14)	C18—C19—H19C	111.3
N1—C1—C2	105.69 (12)	C20—C19—H19C	111.3
N2—C2—C3	124.33 (14)	C18—C19—H19D	111.3
N2—C2—C1	129.33 (13)	C20—C19—H19D	111.3
C3—C2—C1	106.29 (12)	H19C—C19—H19D	109.2
C4—C3—C8	120.65 (15)	C21—C20—C19	97.6 (5)
C4—C3—C2	132.58 (15)	C21—C20—H20C	112.2
C8—C3—C2	106.77 (13)	C19—C20—H20C	112.2
C3—C4—C5	118.04 (17)	C21—C20—H20D	112.2
C3—C4—H4A	121.0	C19—C20—H20D	112.2
C5—C4—H4A	121.0	H20C—C20—H20D	109.8
C4—C5—C6	120.79 (18)	O3—C21—C20	105.2 (8)
C4—C5—H5	119.6	O3—C21—H21C	110.7
C6—C5—H5	119.6	C20—C21—H21C	110.7
C5—C6—C7	121.64 (16)	O3—C21—H21D	110.7
C5—C6—H6A	119.2	C20—C21—H21D	110.7
C7—C6—H6A	119.2	H21C—C21—H21D	108.8
C8—C7—C6	117.22 (16)	C21'—O3'—C18'	108.2 (10)
C8—C7—H7	121.4	O3'—C18'—C19'	103.9 (9)
C6—C7—H7	121.4	O3'—C18'—H18A	111.0
C7—C8—C3	121.65 (15)	C19'—C18'—H18A	111.0
C7—C8—N1	128.92 (15)	O3'—C18'—H18B	111.0
C3—C8—N1	109.42 (13)	C19'—C18'—H18B	111.0
N4—C9—N3	112.67 (13)	H18A—C18'—H18B	109.0
N4—C9—S1	126.85 (11)	C18'—C19'—C20'	100.5 (7)
N3—C9—S1	120.48 (11)	C18'—C19'—H19A	111.7
O2—C10—N6	127.57 (14)	C20'—C19'—H19A	111.7
O2—C10—C11	126.71 (13)	C18'—C19'—H19B	111.7
N6—C10—C11	105.72 (12)	C20'—C19'—H19B	111.7
N5—C11—C12	124.64 (13)	H19A—C19'—H19B	109.4
N5—C11—C10	129.04 (13)	C21'—C20'—C19'	101.2 (8)
C12—C11—C10	106.31 (12)	C21'—C20'—H20A	111.5
C13—C12—C17	120.17 (13)	C19'—C20'—H20A	111.5
C13—C12—C11	132.86 (13)	C21'—C20'—H20B	111.5
C17—C12—C11	106.91 (12)	C19'—C20'—H20B	111.5
C14—C13—C12	118.37 (15)	H20A—C20'—H20B	109.3
C14—C13—H13	120.8	O3'—C21'—C20'	107.5 (9)
C12—C13—H13	120.8	O3'—C21'—H21A	110.2
C13—C14—C15	120.77 (15)	C20'—C21'—H21A	110.2
C13—C14—H14	119.6	O3'—C21'—H21B	110.2
C15—C14—H14	119.6	C20'—C21'—H21B	110.2
C16—C15—C14	121.45 (15)	H21A—C21'—H21B	108.5
C16—C15—H15	119.3		

### Hydrogen-bond geometry (Å, º)

**Table:**

*D*—H···*A*	*D*—H	H···*A*	*D*···*A*	*D*—H···*A*
N1—H1···O3^i^	0.88 (1)	1.96 (2)	2.829 (15)	168 (2)
N1—H1···O3′^i^	0.88 (1)	1.98 (3)	2.84 (2)	167 (2)
N3—H3···O1	0.85 (1)	2.18 (2)	2.8369 (16)	134 (2)
N4—H4···O2	0.86 (1)	2.10 (2)	2.7857 (16)	136 (2)
N6—H6···O1^ii^	0.84 (1)	2.32 (2)	3.0522 (16)	146 (2)

Symmetry codes: (i) *x*, *y*−1, *z*; (ii) *x*, *y*+1, *z*.
